# Loss of WNK1 Suppressed the Malignant Behaviors of Hepatocellular Carcinoma Cells by Promoting Autophagy and Activating AMPK Pathway

**DOI:** 10.1155/2022/6831224

**Published:** 2022-12-30

**Authors:** Fang Wang, Xianxia Yan, Guangyan Shi, Lei Zhang, Xu Jing

**Affiliations:** ^1^College of Life Science, Shandong Normal University, Jinan, 250014 Shandong, China; ^2^Institute of Medical Sciences, The Second Hospital, Cheeloo College of Medicine, Shandong University, Jinan, 250033 Shandong, China; ^3^Clinical Laboratory of Shandong Mental Health Center, China; ^4^Department of Clinical Laboratory, The Second Hospital, Cheeloo College of Medicine, Shandong University, Jinan, 250033 Shandong, China; ^5^Microbiome-X, National Institute of Health Data Science of China & Institute for Medical Dataology, Cheeloo College of Medicine, Shandong University, Jinan, 250002 Shandong, China; ^6^Department of Biostatistics, School of Public Health, Cheeloo College of Medicine, Shandong University, Jinan, 250002 Shandong, China; ^7^Department of Microbiology, Tumor and Cell Biology, Karolinska Institute, 171 65 Stockholm, Sweden

## Abstract

**Background:**

WNK lysine deficient protein kinase 1 (WNK1) has been shown to be highly expressed in hepatocellular carcinoma (HCC) samples and related to poor prognosis of HCC patients based on bioinformatics analysis. However, the specific function of WNK1 in HCC has not been analyzed. This study is aimed at exploring the function of WNK1 in HCC progression as well as its related molecular mechanism.

**Methods:**

After knockdown of WNK1 by small interference RNA, cell counting kit-8, colony formation, western blot, Transwell, and wound healing assays were employed to evaluate the biological behaviors of HCC cells. Immunofluorescent staining was applied to detect the effect of WNK1 on LC3 II. GSK690693 or si-AMPK was applied to block AMPK pathway. The expression of autophagy and AMPK pathway related molecules was examined by western blot assay.

**Results:**

WNK1 was highly expressed in HCC cell lines and loss of WNK1 inhibited HCC cell proliferation, cell cycle, migration, and invasion. Additionally, we demonstrated that loss of WNK1 promoted the autophagy and activated AMPK pathway in HCC cells. While, GSK690693 treatment or si-AMPK transfection suppressed the autophagy and promoted HCC cells proliferation. However, WNK1 knockdown counteracted the effect of GSK690693 or si-AMPK in regulating HCC cell proliferation. Finally, we demonstrated that WNK1 regulated the malignant behaviors of HCC cells by modulating autophagy and AMPK pathway.

**Conclusions:**

The above results indicated that WNK1 may be a worthwhile target to be considered for therapy of HCC.

## 1. Introduction

Hepatocellular carcinoma (HCC) is a malignant tumor generating from liver cells, which is very common in Asia including China, accounting for approximately 80% of all liver cancer types [1, 2]. At present, a growing number of studies have suggested that the occurrence and progression of HCC were associated with the abnormal expression of many oncogenes and the inactivation of cancer suppressor genes, which were increasingly used in the early diagnosis and treatment of HCC [3, 4]. Therefore, it is of great value to explore the target molecules and molecular mechanisms of HCC for the development of more effective diagnosis and treatment strategies.

WNK lysine deficient protein kinase (WNK), as a typical of protein kinases, binds its conserved lysine residues to ATP at different locations in the kinase domain. Among the four mammalian WNK subtypes, WNK1 is the longest and widely expressed [5]. WNK1 knockdown can block migration and angiogenesis in experiments with cultured primary human endothelial cells [6]. In addition to its great value in vascular biology, WNK1 has been presented to promote migration of cells involved in multiple cancer types [7]. For example, *in vitro* and *in vivo* experiments jointly demonstrated that loss of WNK1 significantly reduced migration, invasion, and metastasis in multiple breast cancer models [7]. Moreover, WNK1 could stimulate angiogenesis to accelerate tumor growth and metastasis in colorectal cancer [8]. Additionally, WNK1 can promote renal tumor development by activating TRPC6-NFAT pathway [9]. *In vivo* analysis using mouse and zebrafish models indicated that WNK1 accelerated cancer metastasis in HCC and prostate cancer and performed great values in angiogenesis [10–14]. However, the underlying molecular mechanisms of WNK1 involved in the rapid development and high mortality of HCC remain unclear.

A growing number of evidence suggested that autophagy is significantly activated in tumor cells including HCC, and inducible autophagy generates a strong prosurvival mechanism for cancer cells [15]. A previous study revealed that WNK1 is an inhibitor of autophagy [5]. However, the relationship between WNK1 and autophagy in HCC has not been revealed.

The aim of the study is to explore the expression level of WNK1 in HCC cell lines and the effect of WNK1 depletion in malignant behaviors of HCC cell lines. Moreover, we also detected the function of WNK1 on autophagy and AMPK signaling pathway in HCC cell lines.

## 2. Materials and Methods

Gene expression profiling interactive analysis (GEPIA) is a web-based tool to afford differential expression analysis, profiling plotting, correlation analysis, patients survival analysis based on TCGA, and GTEx data.

### 2.1. Cell Lines and Stimulation

Human HCC cell lines (Huh7, Hep3B, HepG2, and BEL-7402) and normal human hepatocytes L-02 were received from the Chinese Academy of Sciences (Shanghai, China) and cultured in DEME including 10% fetal bovine serum (FBS, Gibco, China) at 37°C in an incubator of 5% CO_2_.

To knockdown WNK1 expression, small interference RNA (siRNA, si-WNK1-1, or si-WNK1-2) against WNK1 was transfected into Huh7 or Hep3B cells, and a scramble siRNA was regarded as a control. GSK690693 (50 nM) or si-AMPK was used to block AMPK signaling pathway.

### 2.2. qPCR

Gene expression of WNK1 was detected in Huh7 and Hep3B cells by qPCR. The whole RNA was extracted using the TRIzol reagent (Invitrogen, USA). The TransScript® One-Step gDNA Removal and cDNA Synthesis SuperMix Complimentary Kit (Applied Biosystems, USA) were applied to generate the cDNA. PCR amplification was carried out on the ABI Prism 7500 Detection System (Applied Biosystems) and fluorescence was monitored in real-time. All the data were normalized to the GAPDH and analyzed using the 2^−*ΔΔ*Ct^ method.

### 2.3. Western Blot

Cell protein was prepared with the support of lysis buffer, and protein concentration was detected using the BCA kit. Then, 30 *μ*g protein was separated by sodium dodecyl sulfate-polyacrylamide gel electrophoresis and then transferred to polyvinylidene fluoride membranes. Next, the membranes were sealed with 5% skimmed milk at ambient temperature for 1 h and then blotted with the following primary antibodies overnight at 4°C: WNK1 (ab137687, 1 : 2000, Abcam), LC3 I/II (ab128025, 1 : 1000, Abcam), Beclin 1 (ab210498, 1 : 1000, Abcam), P62 (ab91526, 1 : 1000, Abcam), AMPK (ab207442, 1 : 1000, Abcam), p-AMPK (ab23875, 1 : 1000, Abcam), and GAPDH (ab181602, 1 : 8000, Abcam). The membranes were blotted with the secondary antibodies for 1 h at ambient temperature. Protein bands were developed with enhanced chemiluminesence and then analysed using Image J software.

### 2.4. Cell Counting Kit-8 Assay

Based on the supplier's direction, cell survival was detected with the help of cell counting kit-8 (Beyotime, China). Huh7 and Hep3B cells were cultured in a 96-well plate with a density of 5 × 10^3^/well. Then, the cells were cultured with 15 *μ*L kit solution, and incubated for 1.5 h, and their absorbance was detected at 450 wave length.

### 2.5. Colony Formation Assay

Cells were sowed into 60 mm dishes and cultured in the medium. The dishes were placed in 37°C incubator for 14 days, and formed colonies were fixed with ethanol and stained with 0.1% crystal violet. The colonies were observed and counted.

### 2.6. Cell Invasion Assay

Cell invasion was evaluated by using Transwell chambers (24-well inserts). In brief, cells (1 × 10^5^) were cultured on the upper chamber in DMEM medium excluding FBS. Then, 600 *μ*L complete culture medium including 12% FBS was put into the lower chamber. After 24 h, the noninvaded cells on the upper surface of the Transwell membrane were removed by using a wet cotton swab. Next, the invaded cells were stained with 0.1% crystal violet and observed under a microscope.

### 2.7. Wound Healing Assay

Cells were sowed into 6-well plates at a density of 1 × 10^5^ cells/well. When the cells were reached 80% confluence, a 100 *μ*L pipette tip was applied to generate a scratch. Subsequently, the widths of the scratch wound were detected at 0 h and 24 h to evaluate cell migration ability.

### 2.8. Immunofluorescent Staining

Huh7 and Hep3B cells, grown on slides, were fixed in methanol, blocked with 10% bovine serum albumin for 10 min, and blotted with the primary antibody against LC3-II at ambient temperature for 1 h. Then, cells were incubated with the secondary antibody at ambient temperature for 1 h. Finally, cells were stained with DAPI for 10 min.

### 2.9. Statistical Analysis

All experimental data were expressed as the mean ± standard deviation of three independent experiments. One-way ANOVA identified significant difference with Bonferroni's post hoc test for three or more groups comparison with GraphPad Prism 8.0. *p* value less than 0.05 was used to represent a statistically significant difference.

## 3. Results

### 3.1. A Significant Increase of WNK1 Was Occurred in HCC Cell Lines and Loss of WNK1 Limited the Proliferative Ability of HCC Cells

**Figure 1:**
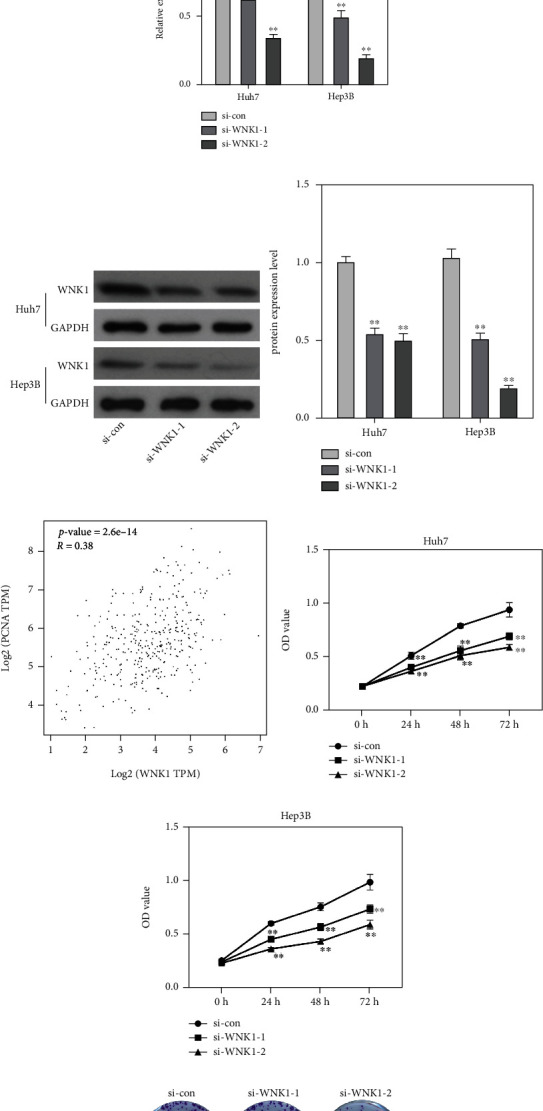
WNK1 was highly expressed in HCC cell lines and promoted cell growth. (a, b) WNK1 expression was significantly increased in HCC cell lines. (c, d) The treatment of si-WNK1 or si-WNK2 obviously reduced WNK1 expression. (e) The relationship between WNK1 and PCNA was analysed by GEPIA website. (f, g) Loss of WNK1 significantly reduced the OD values of Huh7 and Hep3B cells. (h–j) Loss of WNK1 reduced the number of clones in Huh7 and Hep3B cells. ^∗∗^*p* < 0.01 vs. si-con.

We firstly detected WNK1 expression patterns in HCC cell lines, which showed that WNK1 expression was prominently increased in HCC cells including Huh7, HepG2, Hep3B, and BEL-7402 cells compared with the normal cell L-02 (Figures 1(a) and 1(b)). With higher expression level, Huh7 and Hep3B cells were applied for the subsequent experiments. In view of this, siRNA technology was applied to reduce the expression of WNK1 to evaluate the effect of WNK1 depletion on the biological behaviors of HCC cells (Figures 1(c) and 1(d)). Information from GEPIA website suggested that WNK1 expression was positively related to PCNA, insinuating the important function of WNK1 on HCC cells growth (Figure 1(e)). Therefore, CCK-8 and colony formation assays were carried out to detect the function of WNK1 on HCC cells proliferation. We observed that the treatment of si-WNK1-1 or si-WNK1-2 could prominently reduce the OD values of Huh7 and Hep3B cells (Figures 1(f) and 1(g)). Moreover, the number of cell clones was significantly decreased in Huh7 and Hep3B cells after si-WNK1-1 or si-WNK1-2 treatment (Figures 1(h)–1(j)).

### 3.2. Loss of WNK1 Restricted the Cell Cycle, Invasion, and Migration Abilities in HCC Cells

**Figure 2:**
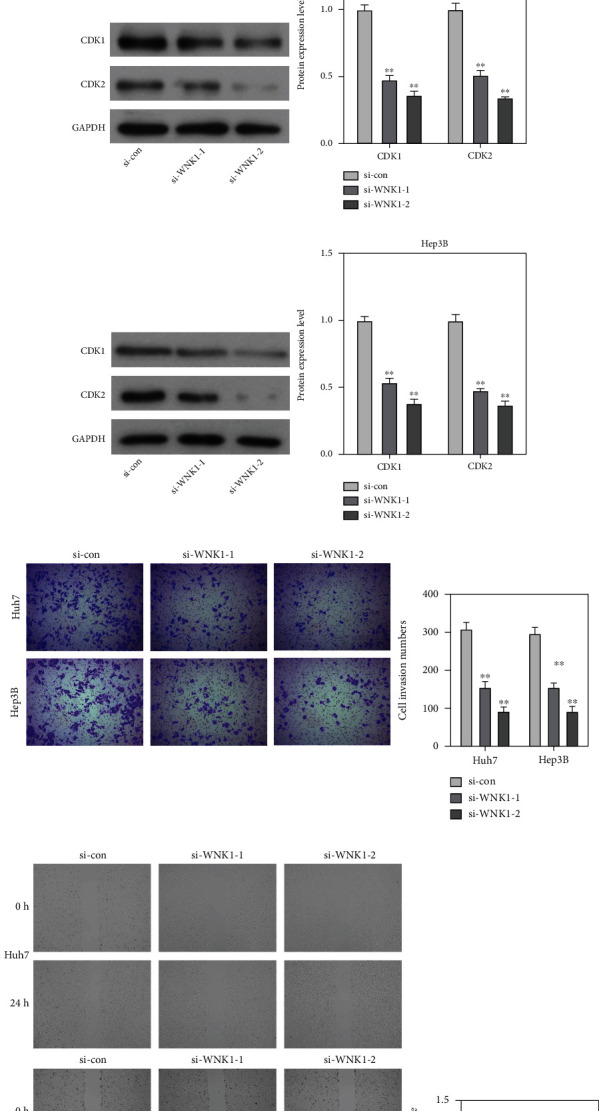
Loss of WNK1 reduced cell cycle, invasion, and migration in HCC cells. (a, b) Data from GEPIA website showed the relationship between WNK1 and CDK1/CDK2. (c, d) Loss of WNK1 reduced the protein patterns of CDK1 and CDK2 in Huh7 and Hep3B cells. (e) Loss of WNK1 decreased the invasion number of Huh7 and Hep3B cells. (f) Loss of WNK1 diminished the migratory area of Huh7 and Hep3B cells. ^∗∗^*p* < 0.01 vs. si-con.

Afterwards, information from GEPIA website showed that WNK1 expression was positively related to CDK1 and CDK2 expression in HCC (Figures 2(a) and 2(b)). Next, western blot was applied to investigate the function of WNK1 on the expression of CDK1 and CDK2 in Huh7 and Hep3B cells. The data from Figures 2(c) and 2(d) showed that loss of WNK1 reduced the expression patterns of CDK1 and CDK2 in Huh7 and Hep3B cells. Additionally, data from Transwell and wound healing assays showed that loss of WNK1 reduced the number of invading cells and the migratory area in Huh7 and Hep3B cells (Figures 2(e) and 2(f)).

### 3.3. Loss of WNK1 Motivated the Autophagy of HCC Cells and Regulated AMPK Pathway

**Figure 3:**
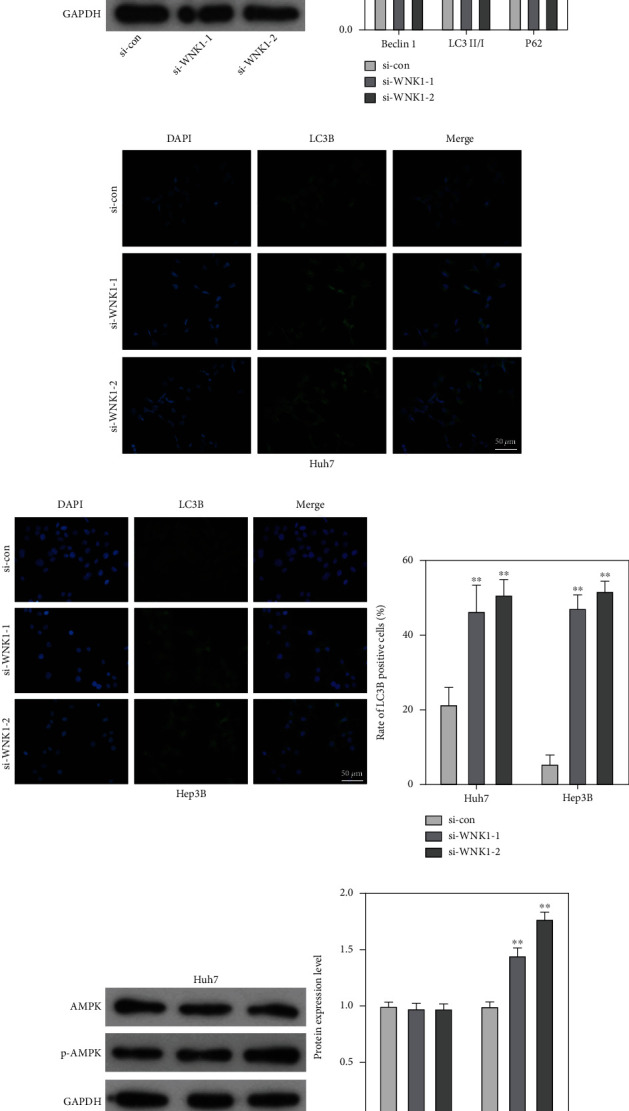
Loss of WNK1 promoted autophagy and AMPK pathway. (a, b) Loss of WNK1 increased the protein patterns of Beclin 1 and LC3 II, and reduced P62 level. (c, d) Immunofluorescence staining was used to detect LC3 II immunoreactivity. (e, f) Loss of WNK1 increased the protein patterns of p-AMPK in Huh7 and Hep3B cells. ^∗∗^*p* < 0.01 vs. si-con.

Data from western blot assay showed that loss of WNK1 obviously increased the pattern of Beclin 1 and the ratio of LC3 II/I, as well as decreased the pattern of P62 in Huh7 and Hep3B cells (Figures 3(a) and 3(b)). Similarly, immunofluorescent staining further confirmed that loss of WNK1 obviously increased the expression of LC3 II compared with that of the control group in Huh7 and Hep3B cells (Figures 3(c) and 3(d)). Considering the importance of AMPK in autophagy process, western blot was applied to detect the function of WNK1 on AMPK pathway. The data showed that loss of WNK1 significantly increased the expression pattern of p-AMPK in Huh7 and Hep3B cells (Figures 3(e) and 3(f)).

### 3.4. Inhibition of AMPK Suppressed the Autophagy and Promoted Cell Growth in HCC Cells

**Figure 4:**
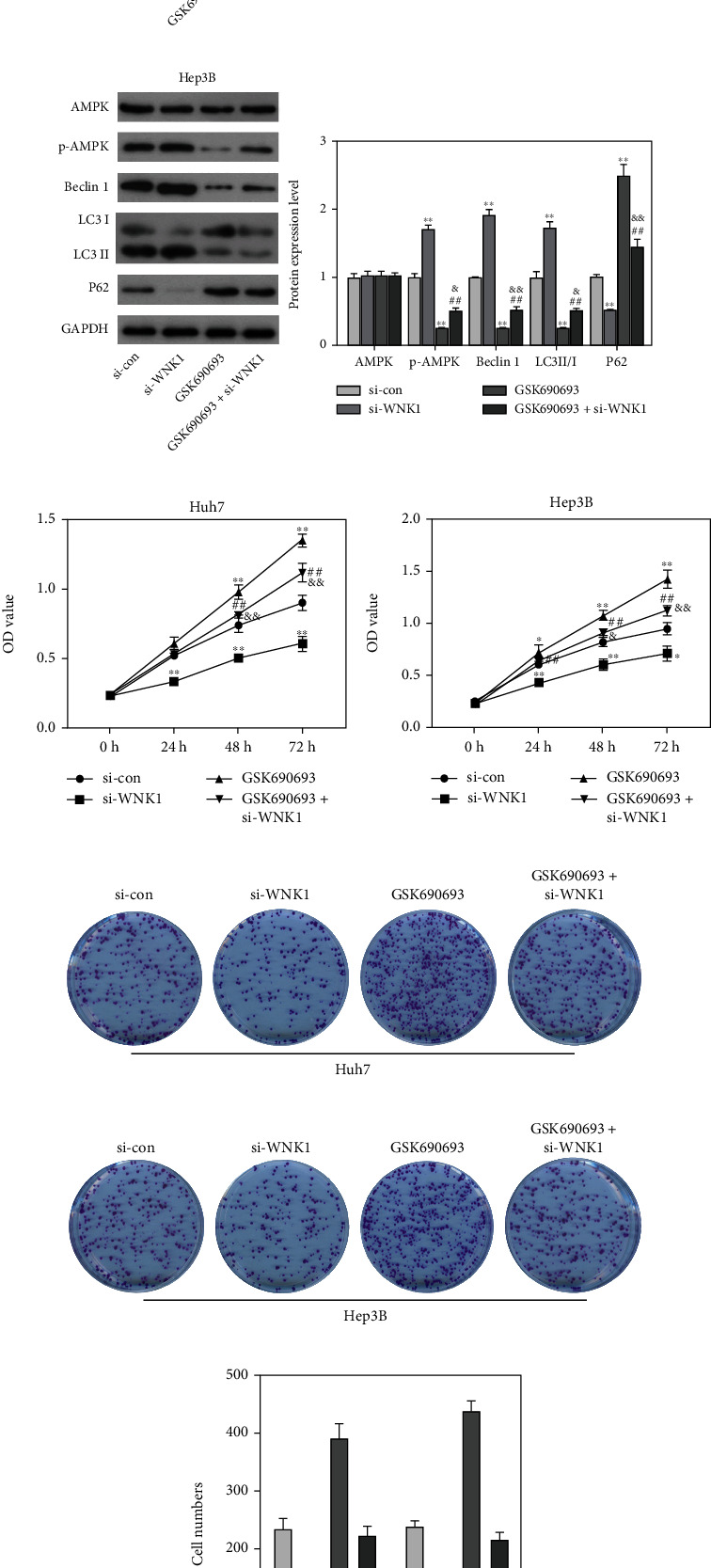
Loss of WNK1 restricted the malignant behaviors of HCC cells by suppressing autophagy and inactivating AMPK pathway. (a, b) GSK690693 treatment reduced the expression patterns of p-AMPK, Beclin 1, LC3 II/I, and increased P62 level, and the above results were reversed after si-WNK1 treatment. (c, d) GSK690693 treatment increased the OD values of Huh7 and Hep3B cells, while si-WNK1 treatment counteracted the effect of GSK690693 on the OD values of Huh7 and Hep3B cells. (e–g) GSK690693 treatment increased the clone number of Huh7 and Hep3B cells, while si-WNK1 treatment counteracted the effect of GSK690693 on the clone number of Huh7 and Hep3B cells. ^∗^*p* < 0.05, ^∗∗^*p* < 0.01 vs. control, ^##^*p* < 0.01 vs. si-WNK1, ^&^*p* < 0.05, ^&&^*p* < 0.01 vs. GSK690693.

To further analyse the function of AMPK in the regulation of WNK1 on autophagy, GSK690693 (AMPK inhibitor) was applied. Data from Figures 4(a) and 4(b) showed that GSK690693 treatment significantly reduced the expression patterns of p-AMPK, Beclin 1, LC3 II/I, and increased the level of P62. However, loss of WNK1 counteracted the effect of GSK690693 treatment on p-AMPK, Beclin 1, LC3 II/I, and P62 levels. Subsequently, data from CCK-8 and colony formation assays showed that GSK690693 treatment significantly increased the OD values and the number of colonies in Huh7 and Hep3B cells (Figures 4(c)–4(g)). However, loss of WNK1 antagonised the effect of GSK690693 treatment on HCC cells growth. The above results showed that loss of WNK1 restricted the malignant behaviors of HCC cells by promoting autophagy and activating AMPK pathway.

**Figure 5:**
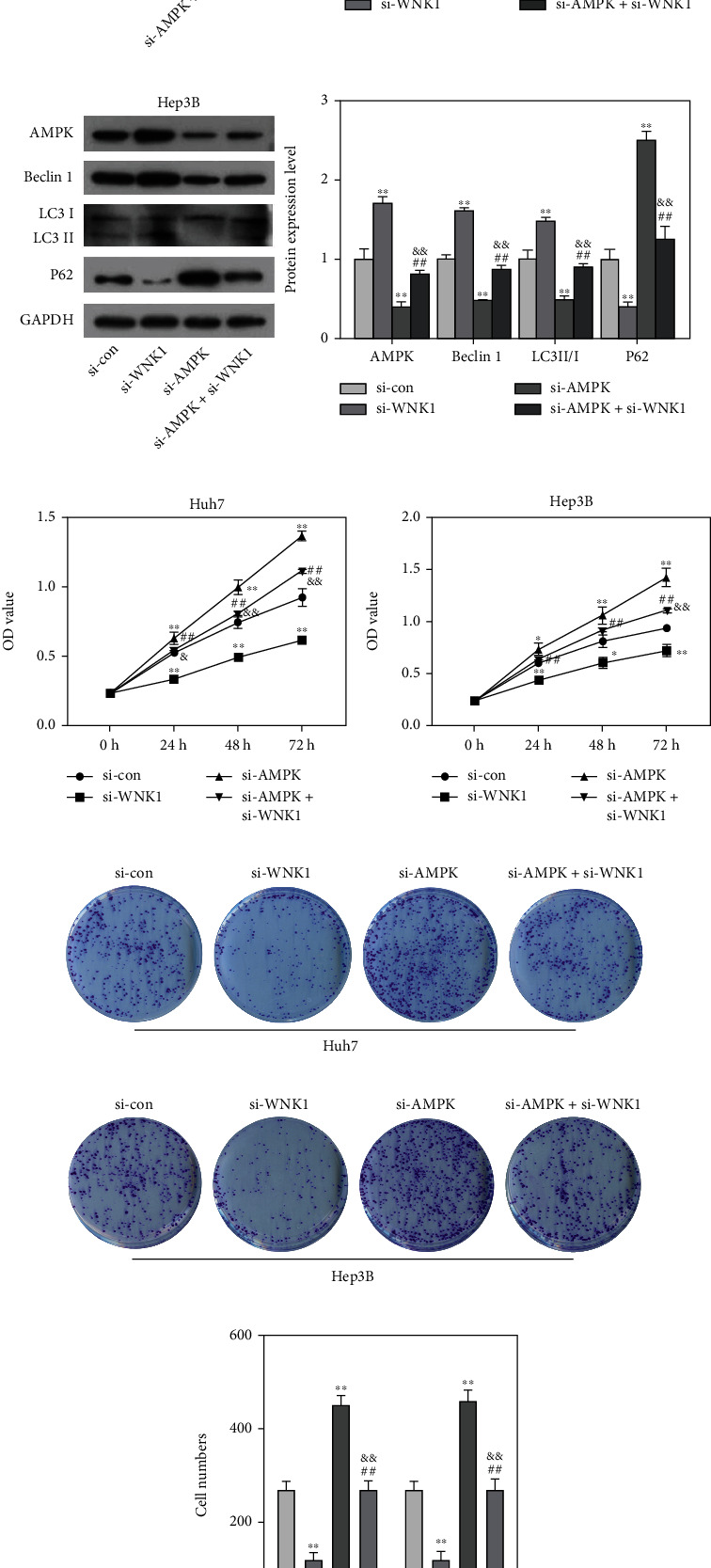
Knockdown of WNK1 suppressed the malignant behaviors of HCC cells by suppressing autophagy and inactivating AMPK pathway. (a, b) si-AMPK transfection reduced the expression patterns of AMPK, Beclin 1, LC3 II/I, and increased P62 level, and the above results were reversed after si-WNK1 treatment. (c, d) si-AMPK transfection increased the OD values of Huh7 and Hep3B cells, while si-WNK1 treatment counteracted the effect of si-AMPK transfection on the OD values of Huh7 and Hep3B cells. (e–g) si-AMPK transfection increased the clone number of Huh7 and Hep3B cells, while si-WNK1 treatment counteracted the effect of si-AMPK transfection on the clone number of Huh7 and Hep3B cells. ^∗^*p* < 0.05, ^∗∗^*p* < 0.01 vs. control, ^##^*p* < 0.01 vs. si-WNK1, ^&^*p* < 0.05, ^&&^*p* < 0.01 vs. si-AMPK.

To further avoid off target effects of AMPK inhibitors, si-AMPK was applied. Data from Figures 5(a) and 5(b) showed that si-AMPK transfection significantly reduced the expression patterns of AMPK, Beclin 1, LC3 II/I, and increased the level of P62. However, loss of WNK1 counteracted the effect of si-AMPK transfection on AMPK, Beclin 1, LC3 II/I, and P62 levels. Subsequently, data from CCK-8 and colony formation assays showed that si-AMPK transfection significantly increased the OD values and the number of colonies in Huh7 and Hep3B cells (Figures 5(c)–5(g)). However, loss of WNK1 antagonised the effect of si-AMPK transfection on HCC cells growth. The above results showed that loss of WNK1 restricted the malignant behaviors of HCC cells by promoting autophagy and activating AMPK pathway.

## 4. Discussion

Previous studies have illustrated that WNK1 may also be a key kinase implicated in various types of cancer [16]. However, the important function of WNK1 in HCC has not been revealed. This problem motivated our research to explore the function of WNK1 in HCC cells. Our study revealed that WNK1 was highly expressed in HCC cell lines, and loss of WNK1 suppressed the malignant behaviors of HCC cells by activating autophagy and AMPK signaling pathway.

Autophagy is an important biological phenomenon conserved from yeast to mammals. Autophagy-related abnormalities are thought to be associated with a variety of human pathologies, from neurodegenerative diseases to cancers, including HCC [17]. Autophagy performs a dual function in cancer, including tumor development and suppression, insinuating that autophagy functioned as a double-edged sword in cancer cells [18]. In HCC, autophagy could promote the cancer development, for example, CCND1 silencing inhibited liver cancer stem cell differentiation by suppressing autophagy [19]. On the other hand, autophagy could block the occurrence of liver-related disease, for example, blocking autophagy and autophagic lysosomal degradation in mice led to hepatosteatosis and hepatomegaly [20], autophagy-deficient mice could develop multiple liver tumors [21], and homeobox containing 1 suppressed liver cancer development by promoting autophagy [22]. The above published reports suggested that autophagy may prevent the development of HCC. A previous study suggested that WNK1 has a universal effect on autophagy and could inhibit the occurrence of autophagy [5]. During autophagy, Beclin 1 takes part in the nucleation of the autophagosomal membrane, and LC3 II is implicated in double-membrane vesicle formation, P62 acts as a substrate to be degraded in the autophagy-lysosome system by binding to ubiquitinated proteins, which are regarded as the biomarkers for autophagy initiation [23, 24]. In our study, we discovered that loss of WNK1 increased the expression of Beclin 1 and LC3 II, as well as reduced P62 expression, suggesting that WNK1 could suppress the autophagy in HCC cells.

AMPK, a serine/threonine protein kinase, functioned as a primary metabolic sensor implicated in cell energy homeostasis [25]. Beyond the energy sensor, the function of AMPK in cancer is still controversial. For example, AMPK was obviously expressed from the occurrence of early hyperplasia to the emergence of large gliomas [26]. Besides that, decreased AMPK signaling in breast cancer patients is compared with strong AMPK signaling in normal breast epithelial cells [27]. Activated AMPK is closely associated with tumor growth, migration, and invasion of cancer cells [28]. AMPK loss could cooperate with oncogenic divers to accelerate tumorigenesis in a tissue-specific manner [28]. Activated AMPK modulated a variety of metabolic processes, including autophagy [29, 30]. Under energy-deprived conditions, AMPK is activated triggering autophagy [31]. In our study, we discovered that loss of WNK1 increased the protein patterns of p-AMPK. However, GSK690693 treatment significantly reduced the levels of p-AMPK, Beclin 1, LC3 II/I, and increased the level of P62. However, loss of WNK1 counteracted the effect of GSK690693 treatment on p-AMPK, Beclin 1, LC3 II/I, and P62 levels. Moreover, GSK690693 treatment significantly increased the OD values and the number of colonies in Huh7 and Hep3B cells, which were inversely after WNK1 depletion.

Some deficiencies should be pointed out. First, the function of WNK1/AMPK was merely confirmed in vitro. Animal experiments for further study will be performed in the further. Second, the upstream regulator of WNK1 and the downstream target of AMPK have not been solved, which should be detected in the future. Third, the LC3II level was measured only in the absence of a lysosomal fusion inhibitor, LC3II/I ratio need to be compared in the presence or absence of lysosome fusion inhibitor. Fourth, other pathways that may be involved in the effect of WNK1 on autophagy have not been elucidated.

## 5. Conclusions

In summary, our data illustrated that WNK1 was highly expressed in HCC cell lines and loss of WNK1 suppressed the proliferation, cell cycle, invasion, and migration of HCC cells. Additionally, we also discovered that loss of WNK1 motivated the autophagy and activated the AMPK pathway. However, GSK690693 treatment or si-AMPK transfection counteracted the activation of autophagy and AMPK pathway by WNK1 depletion.

## Data Availability

The datasets used and/or analyzed during the current study are available from the corresponding author on reasonable request.
